# Supplementary material for: End Sequence Analysis Toolkit (ESAT) expands the extractable information from single-cell RNA-seq data
Source: Genome Res. 2016 Oct;26(10):1397–410. doi: 10.1101/gr.207902.116 (PMC5052061; doi:10.1101/gr.207902.116)
Supplement: Supplemental Material [file supp_gr.207902.116_Supp_Materials.tar.gz › ESAT-esat_v0.1/doc/html/classumms_1_1core_1_1alignment_1_1_feature_sequence_alignment_1_1_unordered_gene_pair.html]

ESAT: umms.core.alignment.FeatureSequenceAlignment.UnorderedGenePair Class Reference


ESAT


- Main Page
- Related Pages
- Packages
- Classes
- Files

- Class List
- Class Index
- Class Hierarchy
- Class Members

All Classes Namespaces Files Functions Variables Enumerator Pages

- umms
- core
- alignment
- FeatureSequenceAlignment
- UnorderedGenePair

Public Member Functions |
List of all members

umms.core.alignment.FeatureSequenceAlignment.UnorderedGenePair Class Reference

Inheritance diagram for umms.core.alignment.FeatureSequenceAlignment.UnorderedGenePair:

[legend]

Collaboration diagram for umms.core.alignment.FeatureSequenceAlignment.UnorderedGenePair:

[legend]

|  |  |
| --- | --- |
| Public Member Functions | |
|  | UnorderedGenePair (Gene gene1, Gene gene2) |
|  | |
| Gene | getFirstGene () |
|  | |
| Gene | getSecondGene () |
|  | |
| int | compareTo (UnorderedGenePair o) |
|  | |
| boolean | equals (Object o) |
|  | |
| int | hashCode () |
|  | |

## Detailed Description

An unordered pair of genes

Author
:   prussell

## Constructor & Destructor Documentation

|  |  |  |  |
| --- | --- | --- | --- |
| umms.core.alignment.FeatureSequenceAlignment.UnorderedGenePair.UnorderedGenePair | ( | Gene | *gene1*, |
|  |  | Gene | *gene2* |
|  | ) |  |  |

Determine which gene is first and which is second

Parameters
:   |  |  |
    | --- | --- |
    | gene1 | Gene 1 |
    | gene2 | Gene 2 |

Here is the call graph for this function:

Here is the caller graph for this function:

## Member Function Documentation

|  |  |  |  |  |  |
| --- | --- | --- | --- | --- | --- |
| int umms.core.alignment.FeatureSequenceAlignment.UnorderedGenePair.compareTo | ( | UnorderedGenePair | *o* | ) |  |

Here is the call graph for this function:

|  |  |  |  |  |  |
| --- | --- | --- | --- | --- | --- |
| boolean umms.core.alignment.FeatureSequenceAlignment.UnorderedGenePair.equals | ( | Object | *o* | ) |  |

Here is the call graph for this function:

|  |  |  |  |  |
| --- | --- | --- | --- | --- |
| Gene umms.core.alignment.FeatureSequenceAlignment.UnorderedGenePair.getFirstGene | ( |  | ) |  |

Returns
:   The first gene

Here is the caller graph for this function:

|  |  |  |  |  |
| --- | --- | --- | --- | --- |
| Gene umms.core.alignment.FeatureSequenceAlignment.UnorderedGenePair.getSecondGene | ( |  | ) |  |

Returns
:   The second gene

|  |  |  |  |  |
| --- | --- | --- | --- | --- |
| int umms.core.alignment.FeatureSequenceAlignment.UnorderedGenePair.hashCode | ( |  | ) |  |

Here is the call graph for this function:

---

The documentation for this class was generated from the following file:

- java/umms/core/alignment/FeatureSequenceAlignment.java


---

Generated on Fri Aug 15 2014 15:11:07 for ESAT by  

 1.8.7
